# Critical Care Nurses’ Perceptions of Abuse and Its Impact on Healthy Work Environments in Five European Countries: A Cross-Sectional Study

**DOI:** 10.3389/ijph.2024.1607026

**Published:** 2024-05-10

**Authors:** Adriano Friganović, Jelena Slijepčević, Slađana Režić, Cristina Alfonso-Arias, Monika Borzuchowska, Anca Constantinescu-Dobra, Madalina-Alexandra Coțiu, Estel Curado-Santos, Beata Dobrowolska, Aleksandra AGutysz-Wojnicka, Maria Hadjibalassi, Mireia Llaurado-Serra, Adrian Sabou, Evanthia Georgiou

**Affiliations:** ^1^ Faculty of Health Studies, University of Rijeka, Rijeka, Croatia; ^2^ University Hospital Centre Zagreb, Zagreb, Croatia; ^3^ University of Applied Health Sciences, Zagreb, Croatia; ^4^ International University of Catalonia, Barcelona, Spain; ^5^ Medical University of Lodz, Łódź, Poland; ^6^ Technical University of Cluj-Napoca, Cluj-Napoca, Romania; ^7^ Granollers General Hospital, Barcelona, Spain; ^8^ Medical University of Lublin, Lublin, Poland; ^9^ University of Warmia and Mazury in Olsztyn, Olsztyn, Poland; ^10^ Cyprus University of Technology, Limassol, Cyprus; ^11^ Cyprus Ministry of Health, Cyprus, Cyprus

**Keywords:** abuse, critical care, nursing, workplace violence, healthy work environment

## Abstract

**Objective:**

Workplace violence is a prevalent phenomenon in hospital settings which critical care nurses are particularly exposed to. The aim of this study was to research abuse against Critical Care Nurses in five European countries, and its association with and impact on Healthy Work Environments.

**Methods:**

This was a multinational cross-sectional study. The 1,183 participants were nurses working in intensive care units from five European countries: Croatia, Cyprus, Poland, Spain, and Romania. The participants were selected by the convenience sampling method from 1 January 2021 to April 2022.

**Results:**

Of 1,033 critical care nurses who answered questions about abuse, 646 reported at least one incident in the previous year. The highest number of incidents came from patients (2,050), followed by another nurse (1,453) and physicians (1,039).

**Conclusion:**

Although nurses in ICUs are aware that a healthy working environment benefits them in their daily work, most of them still face some form of abuse. Organizations must take a realistic approach to prevent abuse and to educate nurses and nurse managers by implementing standards for healthy work environments.

## Introduction

The World Health Organization (WHO) and the International Council of Nurses (ICN) define workplace violence as any incident where staff are abused, threatened or assaulted in circumstances related to their work, including commuting to and from work, involving an explicit or implicit challenge to their safety, wellbeing or health (ILO, ICN, WHO, and PSI, 2002) [1]. No official universal definition of workplace violence or abuse within healthcare settings exists [2].

Violence and abuse are closely related; however, by definition violence is an action which causes destruction, pain, or suffering, while abuse refers to be also prolonged maltreatment that can cause emotional as well as physical trauma [3]. Both terms are used, but in our research we decided on the term “abuse” because it covers a wider group of damaging actions and includes almost any behaviour inflicted by a perpetrator that causes a person distress or harm [4], which relates better to events occurring in intensive care units (ICU). Also, the term abuse was originally in the questionnaire “The AACN Critical Care Nurse Work Environment survey.”

Nurses in ICUs, who are primarily responsible for providing acute life‐saving care to the most vulnerable patients, experience abuse at a significantly higher level than other healthcare professionals [2]. Workplace violence is a prevalent phenomenon in hospital settings, and ICU nurses are particularly exposed to it due to weak points in their work environments [5, 6]. High stress, long hours, a heavy workload and constant pressure from managers and superiors, together with complex care and heightened expectations from patients and families form a basis for creating potential outbursts of any type of abuse. Violence or any form of abuse affects the health and wellbeing of healthcare workers, compromising work performance and job satisfaction [7]. An unhealthy workplace increases the possibility of medical errors, verbal and nonverbal abuse, disrespect, resistance to change, conflicts between healthcare workers and poor provision of care, and also leads to job dissatisfaction and intention to leave the job, which leads to a shortage of nursing staff [8].

According to the American Association of Critical Care Nurses (AACCN), a healthy work environment for nurses is a workplace that is safe, empowering, and satisfying [9]. Additionally, healthy work environments strongly correlate with the psychological health, satisfaction, and wellbeing of nurses [10]. Almost 20 years ago, our colleagues from the AACCN determined six essential standards which, after their implementation in intensive care units, enable a healthier work environment [10]. Intensive care units which have implemented the six standards have seen improvements at all levels, as evidenced by the AACCN data [10, 11]. According to the AACCN, Skilled Communication, True Collaboration, Effective Decision-making, Appropriate Staffing, Meaningful Recognition, and Authentic Leadership are the basic standards which all ICUs need to implement if they want to create a healthier work environment and achieve less moral distress and lower rates of workplace violence [12–14].

After numerous studies of healthy work environments in ICUs, many researchers claim that there is a significant link between the existence of zero-tolerance policies against abuse in hospitals and the amount of verbal and physical abuse which the nurses in their studies experienced [10, 13]. In the available literature there are many articles on the abuse of healthcare workers and the risk factors and risk assessment of abuse, as well as conflict prevention methods and successful management [15–19]. However, we found only one study in Europe and Asia that used the American Healthy Work Environment questionnaire on ICU nurses and linked it to abuse [5]. Despite many findings of abuse against healthcare workers, especially ICU nurses, the prevalence of abuse remains high [4, 5, 17, 20, 21].

Our intention was to explore the perceptions of ICU nurses on abuse in five European countries, the differences in levels of abuse between them, and how much abuse affects healthy work environments. We want to emphasize the importance of a healthy working environment and how necessary it is to try to establish zero tolerance to abuse through hospital policy and nurse managers.

The aim of this study was to explore abuse against critical care nurses in five European countries, and its association with and impact on healthy work environments.

## Methods

### Research Design

This was a multinational cross-sectional study. The 1,183 participants were nurses working in intensive care units in five European countries: Croatia, Cyprus, Poland, Spain, and Romania. The participants were selected by using the convenience sampling method in the period January 2021 to April 2022. A digital questionnaire, with an information part on the first page about the nature of the study, was distributed through the emails of ICU nurses; most of the researchers in this study work in ICUs and have access to their fellow employees’ emails through the websites of the national organizations of each country. Only the researchers from Romania needed to ask head nurses and directors of ICUs to help them distribute the materials; the questionnaire in Romania was delivered both electronically and in printed form, according to the individual preference of each ICU taking part in the study. In the case of Romania, invitation to take part in the study was sent to all hospitals with an ICU in the country. All the Registered Nurses working in adult ICUs from the five countries were eligible to participate. Ethics approval was obtained from the Cyprus Bioethical Committee, as Cyprus was the lead co-ordinating country for the project. The completed questionnaires were digitally and anonymously returned by the participating CCNs simply by answering the final question. Prior the questionnaire informed consent was signed. Each participant registered under a unique code, so our chief statistician could monitor the number of completed questionnaires at any time.

### Participants

The participants were CCNs working in ICUs from five European countries: Croatian, Cyprus, Poland, and Romania. The participants were selected by using the convenience sampling method. From a total of 1,183 respondents to the questionnaire, 1,033 answered questions about abuse, thus meeting the inclusion criteria and making up the sample.

### Questionnaire

The AACN Critical Care Nurse Work Environment survey version 1 was used. The key parts of the Healthy Work Environment scale, based on the six AACN HWE standards, consist of a 32-item survey with 16 individual items, which include ratings of the critical care nurses’ work units and organizations. The scale measures the health of the work environment using Likert-type statements with 4-point response options: strongly disagree (1), disagree (2), agree (3), and strongly agree (4). Since this was a survey, we did translation/back translation and reliability internal consistency of the scale check using Cronbach’s alpha coefficient 0.78 to 0.97.

The questionnaire comprises four sections: Section A has 6 questions related to knowledge of the healthy working environment standards; Section B has 16 questions related to respondents’ attitudes towards the HWE standards; Section C has 20 questions related to managers’ communication and cooperation skills, and questions related to undesirable professional behaviour such as abuse (verbal, physical, sexual) and ways to react in these situations. Section D contains 8 questions referring to demographic data such as gender, age and education level. For this study we added a demographic section, and we adjusted section C so that it was better adapted to nurses from Europe. In agreement with the AACCN we did not change the questions in Section B.

### Data Analysis

The data was analysed using the SPSS R Version 4.1.0, R Core Team (2021), with the following statistical analyses: descriptive statistics, chi-square tests of association, Pearson’s chi-squared test and the Welch two sample t-test for correlation. For categorical variables, only frequencies and percentages of respondents were shown. Chi-squared test was used to analyse the association between categorical variables, mainly differences between countries. For the description of nurse managers’ skills, Mean and Standard Deviation was used, and statistical significance of differences between 2 groups was calculated by T-test for independent samples. All differences that had *p* < 0.05 were considered statistically significant.

## Results

A total of 1,033 respondents from the five countries participated in the study: Croatia *n* = 257, Cyprus *n* = 226, Poland *n* = 75, Spain *n* = 232, Romania *n* = 243. Most of the respondents were women (69.3%). With regard to the age of the respondents, the largest group in the total sample consisted of 20–35-year-olds, totalling 443 respondents (42.9%). In terms of years of work experience in the ICU, although the largest group of respondents (38.4%) had 0–5 years of work experience, the difference with respect to the other two categories was small. Most respondents worked in a general ICU (60.9%). This data is presented in Table 1.

**TABLE 1:** Demographic data from respondents who answered questions related to abuse, Improving Working Environments for Nurses in the Critical Care Unit Cyprus, Croatia, Poland, Spain, Romania (2019–2022).

		Croatia *N* = 257	%	Cyprus *N* = 226	%	Poland *N* = 75	%	Spain *N* = 232	%	Romania *N* = 243	%	Overall *N* = 1,033	%
Gender	Male	**62**	24.1	**67**	29.6	**7**	9.3	**40**	17.2	**22**	9.0	**198**	19.1
	Female	**172**	66.9	**112**	49.5	**65**	86.7	**189**	81.5	**178**	73.3	**716**	69.3
	Prefer not to answer	**23**	8.9	**47**	20.8	**3**	4.0	**3**	1.3	**43**	17.7	**119**	11.6
Age	20–35	**166**	64.6	**123**	54.4	**23**	30.7	**93**	40.1	**38**	15.7	**443**	42.9
	36–50	**74**	28.8	**56**	24.8	**34**	45.3	**92**	39.6	**131**	53.9	**387**	37.5
	51–65	**17**	6.6	**47**	20.8	**18**	24.0	**47**	20.3	**74**	30.4	**203**	19.6
Years of nursing experience in ICU	0–5	**128**	49.8	**96**	42.5	**25**	33.3	**82**	35.3	**65**	26.7	**396**	38.4
	6–15	**70**	27.2	**57**	25.2	**26**	34.7	**73**	31.5	**76**	31.3	**302**	29.2
	16–40	**59**	22.9	**73**	32.3	**24**	32.0	**77**	33.2	**102**	42.0	**335**	32.4
Type of ICU	General	**61**	23.7	**148**	65.5	**55**	73.3	**162**	69.8	**204**	84.0	**630**	60.9
	Cardio-neuro surgical	**158**	61.5	**14**	6.2	**17**	22.7	**54**	23.3	**36**	14.8	**279**	27.1
	Other	**38**	14.8	**64**	28.3	**3**	4.0	**16**	6.9	**3**	1.2	**124**	12.0

Of the 1,033 critical care nurses who answered the questions about abuse, 646 (Croatia *n* = 145, Cyprus *n* = 133, Poland *n* = 63, Spain *n* = 133, Romania *n* = 172) reported at least one incident in the previous year (harassment/verbal or physical abuse) (Table 2).

**TABLE 2:** Percentage of abuse in the past year, by type and perpetrator—all countries, Improving Working Environments for Nurses in the Critical Care Unit Cyprus, Croatia, Poland, Spain, Romania (2019–2022).

Perpetrator	Verbal abuse—%					Harassment—%					Physical abuse—%				
	Cy	Sp	Cro	Ro	Pol	Cy	Sp	Cro	Ro	Pol	Cy	Sp	Cro	Ro	Pol
Patient	13.2	19.8	43.9	45.6	58.6	1.7	2.5	4.2	0.0	4.0	5.3	6.0	13.6	10.2	50.6
Another nurse	11.5	10.3	36.5	20.1	56.0	1.7	1.2	0.7	0.3	1.3	2.2	0.4	1.1	0.3	4.0
Physician	10.6	13.3	33.4	24.2	52.0	1.3	0.8	1.9	0.0	4.0	0.8	1.2	1.1	0.3	4.0
Nurse manager	6.6	5.6	22.5	9.0	46.6	0.4	0.0	0.0	0.0	0.0	0.4	0.0	1.1	0.0	0.0
Patient’s family	14.6	15.9	24.1	30.4	50.6	0.4	0.4	0.7	0.0	0.0	0.8	1.2	0.7	0.3	9.3
Other healthcare personnel	2.6	3.8	14.7	6.9	12.0	0.8	0.4	0.0	0.3	0.0	0.4	0.8	0.0	0.3	0.0
Administrator	3.0	6.8	10.5	0.8	17.3	0.4	0.4	0.0	0.0	0.0	0.4	0.8	0.0	0.0	0.0

*Cy, Cyprus; Sp, Spain; Cro, Croatia; Ro, Romania; Pol, Poland.

The highest number of incidents is coming from patients (2,050), followed by another nurse (1,453) and physicians (1,039) (Table 2).

In answer to the question “Have you reported the incidents?”, 294 (46%) said they did not report any of the incidents that occurred, while 352 (54%) reported at least some of them, and 191 (30%) reported all of them.

Of the respondents who reported the incident(s) (or some of them), 43% reported that subsequently there was some discussion, but nothing was done or there was no follow up. There were significant differences across the countries (*p* < 0.001). In Cyprus (the highest), this type of response was indicated by 66% of the RNs, and in Spain (the lowest) by 33%.

We wanted to determine whether there was an association between the items “What happened when you reported the incident(s)?” and “My organization values my health and safety.” There was a significantly higher level of agreement (Pearson’s Chi-squared test <0.001) with the statement “The organization values my health and safety” among those respondents who reported that the incidents were resolved in a satisfactory manner, than among those respondents who were blamed for the incident or those who specified some other turn of event.

When asked if their organization has a zero-tolerance policy on verbal abuse, the respondents answered yes in the smallest percentage in Poland (17%), and in the highest percentage in Croatia and Romania (33%). It is interesting that the largest percentage of respondents in all countries except Poland answered that they did not know if their organization had a zero-tolerance policy. Similar results were obtained when asked if their organization has a zero-tolerance policy on physical abuse. 50% of respondents in Croatia answered yes, while in Cyprus only 28% answered yes (Table 3).

**TABLE 3:** Answers of participants about their organization’s zero-tolerance policy on verbal/physical abuse, Improving Working Environments for Nurses in the Critical Care Unit Cyprus, Croatia, Poland, Spain, Romania (2019–2022).

		Overall, *N* = 1,033^a^	Cyprus, *N* = 194^a^	Spain, *N* = 232^a^	Croatia, *N* = 257^a^	Poland, *N* = 75^a^	Romania, *N* = 275^a^	Chi-square *p*-value^b^
		N (%)	N (%)	N (%)	N (%)	N (%)	N (%)	
Does your organization have a zero tolerance policy on verbal abuse?	Yes	287 (28%)	31 (16%)	66 (28%)	85 (33%)	13 (17%)	92 (33%)	<0.001
	No	279 (27%)	60 (31%)	39 (17%)	59 (23%)	38 (51%)	83 (30%)	
	Don’t know	467 (45%)	103 (53%)	127 (55%)	113 (44%)	24 (32%)	100 (36%)	
Does your organization have a zero tolerance policy on physical abuse?	Yes	432 (42%)	54 (28%)	110 (47%)	128 (50%)	30 (40%)	110 (40%)	
	No	146 (14%)	29 (15%)	17 (7.3%)	25 (9.7%)	17 (23%)	58 (21%)	<0.001
	Don’t know	455 (44%)	111 (57%)	105 (45%)	104 (40%)	28 (37%)	107 (39%)	

^a^ n (%).

^b^ Pearson’s Chi-squared test.

An association was determined between the questions “Does your organization have a zero-tolerance policy on physical abuse?”, and “How would you rate the quality of communication in your unit among the following?”. Nurses who agreed with the statement that their organization has a zero-tolerance policy to physical abuse rated the communication between nurses (0.002), as well as between nurses and unit nurse managers (<0.001) and nurses and hospital administration (<0.001), as significantly better than the other two subgroups (those who thought that their organization does not have a zero-tolerance policy to physical abuse and those who did not know). Communication between nurses and physicians (<0.001) was perceived as being better among the nurses who stated that there is a zero-tolerance policy in their organization than among the nurses who did not know whether there is a zero-tolerance policy or not.

An association was determined between the questions “Does your organization have a zero-tolerance policy on physical abuse?”, and “How would you rate the quality of collaboration in your unit among the following?”. Nurses who stated that their organization has a zero-tolerance policy to physical abuse significantly more often considered collaboration between nurses (<0.001), and between nurses and physicians (<0.001), to be excellent, compared to the other two subgroups (those who thought their organization does not have zero tolerance policy to physical abuse and those who did not know). Collaboration between nurses and unit nurse managers (<0.001), as well as between nurses and the hospital administration (<0.001), was perceived as being significantly better by nurses who stated that their organization has a zero-tolerance policy to physical abuse, than by the other two subgroups (those who thought their organization does not have zero tolerance policy on physical abuse and those who did not know).

An association was determined between the questions “Does your organization have a zero-tolerance policy on physical abuse?”, and “In your unit how would you rate the respect for nurses by each of the following?”. Respect for nurses by other nurses (<0.001) was perceived as excellent by statistically more nurses who stated that there was a zero-tolerance policy on physical abuse in their organization, than by those who said there was not, or who did not know. Physicians’ respect (0.019) and respect by the hospital administration (<0.001) was perceived as being better by nurses who stated that there was a zero-tolerance policy on physical abuse in their organization, compared to the other two subgroups. Nurses who had a zero-tolerance policy on physical abuse in their organization experienced significantly higher levels of respect from other healthcare colleagues (<0.001) and unit nurse managers (<0.001), than nurses who did not think their organization has a zero-tolerance policy.

An association was determined between the questions “Does your organization have a zero-tolerance policy on physical abuse?”, and “Please rate the skill of your unit nurse managers in the following areas.” Nurses who stated that there is a zero-tolerance policy rated their unit’s nurse managers as statistically significantly better than the other two subgroups (those who did not have a zero-tolerance policy in their organizations and those who did not know) on all the evaluated aspects: communication; collaboration; providing staff resources; providing supplies, equipment, and other non-human resources; effective decision-making; recognition of others’ contribution; leadership; ensuring the provision of high quality patient care; promoting a professional practice environment; and overall effectiveness (<0.001).

Table 4 shows the mean level [SD] of the rating of the manager’s skills across the groups of nurses who had experienced at least one incident of abuse. Higher means score in Table 4 shows better skill for nurse manager. Nurses who had experienced verbal abuse from a nurse manager evaluated all their nurse managers’ skills as significantly lower than nurses who had not experienced verbal abuse in the past year. Nurses who had experienced physical abuse from a nurse manager (*N* = 6) evaluated their nurse managers significantly lower than nurses who had not experienced that type of abuse in many skills: proving staff resources; providing supplies, equipment and other non-human resources; effective decision-making; leadership; promoting a professional practice environment; overall effectiveness; and ensuring the provision of high-quality patient care. Nurses who had experienced sexual harassment from nurse managers tended to give lower evaluations of managers’ skills, with significantly lower evaluations of managers’ communication, providing staff resources, providing supplies, equipment, and other non-human resources, ensuring the provision of high-quality patient care, promoting a professional practice environment, and overall effectiveness.

**TABLE 4:** Abuse incidents by nurse managers vs. managers’ skills, Improving Working Environments for Nurses in the Critical Care Unit Cyprus, Croatia, Poland, Spain, Romania (2019–2022).

Please rate the skill of your unit nurse managers in the following areas	Any incident of abuse from a nurse manager								
	Verbal abuse			Physical abuse			Sexual harassment		
	No, *N* = 526^a^	Yes, *N* = 120^a^	*p*-value^b^	No, *N* = 640^a^	Yes, *N* = 6^a^	*p*-value^b^	No, *N* = 642^a^	Yes, *N* = 4^a^	T-test *p*-value^b^
	M (SD)	M (SD)	M (SD)	M (SD)	M (SD)	M (SD)	M (SD)	M (SD)	
Communication	2.8 (0.9)	2.0 (0.9)	<0.001	2.7 (1.0)	2.2 (1.5)	0.4	2.7 (1.0)	2.0 (0.0)	<0.001
Collaboration	2.9 (0.9)	2.0 (0.9)	<0.001	2.7 (1.0)	2.2 (1.5)	0.4	2.7 (1.0)	2.2 (0.5)	0.14
Proving staff resources	2.5 (1.0)	1.9 (0.8)	<0.001	2.4 (1.0)	1.7 (0.5)	0.021	2.4 (1.0)	1.3 (0.5)	0.020
Providing supplies, equipment, and other non-human resources	3.0 (0.8)	2.4 (0.8)	<0.001	2.9 (0.9)	1.7 (0.5)	0.002	2.9 (0.9)	3.0 (0.0)	<0.001
Effective decision-making	2.8 (0.9)	2.0 (0.8)	<0.001	2.7 (1.0)	1.2 (0.4)	<0.001	2.6 (1.0)	2.2 (0.5)	0.2
Recognition of others’ contribution	2.7 (1.0)	1.8 (0.9)	<0.001	2.5 (1.0)	1.7 (0.8)	0.052	2.5 (1.0)	1.5 (1.0)	0.14
Leadership	2.7 (1.0)	2.0 (1.0)	<0.001	2.6 (1.0)	1.7 (0.8)	0.039	2.6 (1.0)	1.5 (1.0)	0.12
Ensuring the provision of high-quality patient care	2.9 (0.9)	2.3 (0.9)	<0.001	2.8 (0.9)	1.8 (0.8)	0.024	2.8 (0.9)	1.3 (0.5)	0.008
Promoting a professional practice environment	2.8 (1.0)	2.0 (0.9)	<0.001	2.7 (1.0)	1.8 (0.4)	0.003	2.7 (1.0)	1.3 (0.5)	0.010
Overall effectiveness	2.8 (0.9)	2.1 (0.8)	<0.001	2.7 (0.9)	1.3 (0.5)	0.001	2.7 (0.9)	1.3 (0.5)	0.009

^a^ Mean (SD).

^b^ Welch Two Sample t-test.

## Discussion

Our study showed that nurses in critical care units are exposed to workplace abuse; 62.5% of them had experienced at least one incident in the past year. This is not a surprising finding, because other authors have obtained similar data [18–23]. In their study, Cheraghi et al. found that 74.1% of nurses had been exposed to some form of abuse similar to the results from our study [18]. Fahimeh et al. found that 68.3% of nurses had experienced violent behaviour at their workplace [24], and Roche et al. stated that as many as 80.3% of nurses had experienced some form of abuse during their last five shifts [21]. Georgiu et al. found that proposed blended training program may be used by trainers, who can enable nurses develop the competencies required to influence their work environment, in a context of shared responsibility [22]. In their systematic review, Liu et al., investigating the prevalence rate of violence against healthcare workers in the workplace by patients and family members, concluded that nurses and physicians are the most vulnerable groups, and that in total, from all the studies included in the analysis (253, with a total number of 331,544 participants), 61.9% of the participants stated that they had been exposed to some form of violence in the workplace which is in concordance with results of this study [20].

In 2005, the American Association of Critical Care Nurses published six standards for establishing and maintaining a healthy working environment, the second edition of which was published in 2016. Studies were conducted in 2006, 2008, 2013, 2018 and 2021, which refer to the state of the work environment, and based on the results, propose measures for improvement. The results of the 2021 study show that of the total number of nurses who responded to the online questionnaire (7,399), as many as 5,334 (72%) reported at least one incident of abuse in the past year [10].

Violtence in the workplace has consequences for the work of nurses. Fahimeh et al. state in their research that abuse negatively affects the quality of the working life of nurses; a significant negative correlation was obtained between abuse and the quality of nurses’ working lives (*p* = 0.01, r = −0.173) [24]. Some authors highlight that the state of the work environment is related to the violence experienced by nurses. Roche et al. believe that nurse managers should direct interventions to improve the working environment [21, 25, 26].

Cheraghi et al. state that a large number of nurses are dissatisfied with the management of violence in their institution and suggest interventions such as violence management strategies and zero tolerance for any form of violence [18]. Some other authors state that human resource management is important in violence management strategies and suggest regular staff training programmes for working with aggressive patients, as well as support programmes for people who experience some form of violence [15, 24, 27, 28].

The data of our study, which states that only 28% of nurses from all five countries answered that there is zero tolerance for verbal abuse in their institutions, also shows that violence management strategies should be improved. There are slightly better data when talking about zero tolerance for physical abuse; 42% of the total number of nurses answered that their institution has a zero-tolerance policy on physical abuse.

### Limitations

The major limitation of the current study is the convenience sample that does not allow the generalisation of the findings. The potential bias in this study was that researchers belong to the study population and performed recruitment. This weakness has to be discussed in a prevalence study, as the prevalence might be overestimated. Furthermore, the study has been performed during the COVID-19 pandemic. There is a high probability that abuse incidences were driven in this high workload period on ICUs, and for future research it would be interesting to repeat the study after COVID-19 period with reduced biases.

### Conclusion

Although nurses in ICUs are aware that a healthy working environment benefits them in their daily work, most of them still face some form of abuse. Based on the American example of the implementation of healthy work environment standards, it is time to create and sustain healthy work environments that lead to more satisfied nurses. Organizations must take a realistic approach to stop abuse, and to educate nurses and nurse managers by implementing standards for healthy work environments. Fostering a healthy work environment takes continual effort, and it is necessary to begin it as soon as possible in each European country. Future recommendation is urgent implementation of healthy work environments standards in healthcare institutions.
