# Corrigendum: Critical Care Nurses’ Perceptions of Abuse and Its Impact on Healthy Work Environments in Five European Countries: A Cross-Sectional Study

**DOI:** 10.3389/ijph.2024.1607849

**Published:** 2024-08-29

**Authors:** Adriano Friganović, Jelena Slijepčević, Slađana Režić, Cristina Alfonso-Arias, Monika Borzuchowska, Anca Constantinescu-Dobra, Madalina-Alexandra Coțiu, Estel Curado-Santos, Beata Dobrowolska, Aleksandra AGutysz-Wojnicka, Maria Hadjibalassi, Mireia Llaurado-Serra, Adrian Sabou, Evanthia Georgiou

**Affiliations:** ^1^ Faculty of Health Studies, University of Rijeka, Rijeka, Croatia; ^2^ University Hospital Centre Zagreb, Zagreb, Croatia; ^3^ University of Applied Health Sciences, Zagreb, Croatia; ^4^ International University of Catalonia, Barcelona, Spain; ^5^ Medical University of Lodz, Łódź, Poland; ^6^ Technical University of Cluj-Napoca, Cluj-Napoca, Romania; ^7^ Granollers General Hospital, Barcelona, Spain; ^8^ Medical University of Lublin, Lublin, Poland; ^9^ University of Warmia and Mazury in Olsztyn, Olsztyn, Poland; ^10^ Cyprus University of Technology, Limassol, Cyprus; ^11^ Cyprus Ministry of Health, Cyprus, Cyprus

**Keywords:** abuse, critical care, nursing, workplace violence, healthy work environment

An author name was incorrectly spelled as “Cristina Cristina Alfonso-Arias”. The correct spelling is “Cristina Alfonso-Arias”. An author name was incorrectly spelled as “Estel Estel Curado-Santos”. The correct spelling is “Estel Curado-Santos”. An author name was incorrectly spelled as “Mireia Laurado-Serra”. The correct spelling is “Mireia Llaurado-Serra”.

The authors apologize for these errors and state that this does not change the scientific conclusions of the article in any way. The original article has been updated.

